# Do P-glycoprotein-mediated drug-drug interactions at the blood-brain barrier impact morphine brain distribution?

**DOI:** 10.1007/s10928-024-09957-0

**Published:** 2025-01-07

**Authors:** Berfin Gülave, Ariel Lesmana, Elizabeth CM de Lange, JG Coen van Hasselt

**Affiliations:** Division of Systems Pharmacology and Pharmacy, Leiden Academic Center for Drug Research, Leiden University, Einsteinweg 55, Leiden, 2333 CC The Netherlands

**Keywords:** P-glycoprotein, Blood-brain barrier, Drug-drug interaction, Physiologically-based pharmacokinetics (PBPK), LeiCNS-PK3.0, Rowland and Matin equation

## Abstract

P-glycoprotein (P-gp) is a key efflux transporter and may be involved in drug-drug interactions (DDIs) at the blood-brain barrier (BBB), which could lead to changes in central nervous system (CNS) drug exposure. Morphine is a P-gp substrate and therefore a potential victim drug for P-gp mediated DDIs. It is however unclear if P-gp inhibitors can induce clinically relevant changes in morphine CNS exposure. Here, we used a physiologically-based pharmacokinetic (PBPK) model-based approach to evaluate the potential impact of DDIs on BBB transport of morphine by clinically relevant P-gp inhibitor drugs.

The LeiCNS-PK3.0 PBPK model was used to simulate morphine distribution at the brain extracellular fluid (brain_ECF_) for different clinical intravenous dosing regimens of morphine, alone or in combination with a P-gp inhibitor. We included 34 commonly used P-gp inhibitor drugs, with inhibitory constants and expected clinical P-gp inhibitor concentrations derived from literature. The DDI impact was evaluated by the change in brain_ECF_ exposure for morphine alone or in combination with different inhibitors. Our analysis demonstrated that P-gp inhibitors had a negligible effect on morphine brain_ECF_ exposure in the majority of simulated population, caused by low P-gp inhibition. Sensitivity analyses showed neither major effects of increasing the inhibitory concentration nor changing the inhibitory constant on morphine brain_ECF_ exposure. In conclusion, P-gp mediated DDIs on morphine BBB transport for the evaluated P-gp inhibitors are unlikely to induce meaningful changes in clinically relevant morphine CNS exposure. The developed CNS PBPK modeling approach provides a general approach for evaluating BBB transporter DDIs in humans.

**Supplementary Information:**

The online version contains supplementary material available at 10.1007/s10928-024-09957-0.

## Introduction

Drug-drug interactions (DDI) can lead to changes in pharmacokinetic (PK) and/or pharmacodynamic (PD) relationships [1, 2]. DDIs at the PK level may arise from modulation of metabolizing enzymes and/or active transporters located at physiological barriers. By inhibiting or inducing enzymes or transporters, drug concentrations in plasma or tissue compartments can be altered, which may lead to an increased risk for toxicities or reduced efficacy. Therefore, characterization of DDIs is of high clinical relevance [3, 4].

Transporter-mediated DDIs have received increasing attention due to developing insights into their important role drug disposition [1, 5]. In this context, P-glycoprotein (P-gp) is an important and extensively studied transporter, as it is involved in membrane transport processes for many drugs. Drugs that induce or inhibit P-gp can interact with other P-gp substrates drugs leading to inhibition of transporter activity and consequently cause DDIs [6, 7]. P-gp is an important efflux transporter at the BBB and may therefore contribute to P-gp mediated DDIs that could influence CNS drug exposure. For example, inhibition of P-gp by tariquidar has been shown to increase brain verapamil levels, as observed by positron emission tomography (PET) studies [8]. The same was observed when P-gp was inhibited by cyclosporine [9]. Both studies illustrate the potential impact of DDIs for P-gp mediated transport at the level of the BBB in humans and therefore needs more attention.

Morphine is among the most commonly used opioids for treatment of acute and chronic pain, and also a P-gp substrate. To achieve its effects, morphine needs to cross the blood brain barrier (BBB). BBB transport of morphine is governed by passive para- and transcellular diffusion, as well as by active influx and efflux transporters. The active influx transporter is yet unidentified, and is saturable at relatively low morphine plasma concentrations [10, 11]. The efflux transporters involve P-gp and a probenecid sensitive transporter [10–14]. The influx transporter is mainly dominating at low concentrations and gets saturated at higher concentrations indicating nonlinear transport of morphine across the BBB. This could mean that inhibiting the efflux transporter at low morphine concentrations can have relatively reduced impact compared to increased morphine concentrations where the efflux transporters would be more dominant in morphine transport at BBB. Since morphine is a P-gp substrate, it may be subjected to DDIs at the BBB [15]. This is supported by in vitro and preclinical in vivo studies indicated that morphine is transported by P-gp out of the brain capillary endothelium and that the BBB permeability of morphine may be altered in the presence of P-gp inhibitors [16, 17].

The clinical significance of P-gp mediated DDIs on morphine CNS exposure is however unclear. Conducting clinical DDI studies to assess changes in CNS drug exposure is ethically challenging. To this end, physiologically-based pharmacokinetic (PBPK) modeling represents a useful tool to integrate available preclinical and clinical knowledge to generate predictions on the effect of DDIs and have been used extensively to predict the effect of DDIs on systemic drug exposure. However, the use of PBPK modeling approaches to predict DDIs at the level of the CNS has so far not been explored. Here, we apply our previously developed comprehensive LeiCNS-PK3.0 PBPK model (LeiCNS-PK3.0), which includes nonlinear BBB transport, to evaluate to predict P-gp mediated DDIs at the BBB effects on brain_ECF_ exposure of morphine in humans for clinically relevant P-gp inhibitors.

## Methods

### LeiCNS-PK3.0 with nonlinear BBB transport model implementation

For our simulations, the previously published LeiCNS-PK3.0 with an extension for morphine’s nonlinear BBB transport was used [18]. Briefly, this CNS PBPK model can predict the distribution of small molecules in various CNS and cerebrospinal fluid (CSF) compartments. The model includes BBB and blood-CSF-barrier (BCSFB) characteristics and transport across these barriers [19].

A previously published one-compartmental population model for plasma PK of morphine and its metabolites morphine-3-glucuronide (M3G) and morphine-6-glucuronide (M6G) was used to describe the plasma PK of morphine as input for the CNS PBPK model (Table 1) [20].

Physico-chemical parameters for morphine such as the molecular weight, the partition coefficient LogP, acid and base ionization constants were obtained from DrugBank version 5.0 [21] (Table 1), which were used as input in the LeiCNS-PK3.0 model.

**Table 1:** Plasma pharmacokinetic parameters and physic-chemical properties of morphine

Plasma PK [20]	Morphine
Central clearance (L/hr)	91.9
Central compartment volume (L)	278
Fraction formed^a^	0.323
IIV central clearance (as variance)	0.222
IIV central compartment volume (as variance)	0.747
Proportional residual error (as variance)	0.286
**Drug properties** [21]	
Molecular weight (g/mol)	285.34
Partition coefficient LogP	0.99
Acid ionization constant	10.26
Base ionization constant	9.12
Fraction unbound plasma	0.65
fAFBBB	0.21815^b^
K_p, uu, BBB_^c^	(14.543*Css, u,plasma^− 0.895^)/(5.902^− 0.895^ + Css, u,plasma^− 0.895^)

a fraction of morphine clearance forming metabolites

b obtained from [22–26]

c extent of morphine transport function describing passive diffusion, active efflux and saturable influx transport

fAFBBB asymmetry factor at the blood brain barrier used for translation of rat K_p, uu, BBB_ to human

IIV inter individual variability

### Plasma concentrations for P-gp inhibitors

In total 34 inhibitors (33 clinically used and 1 positive control; tariquidar) were included. From these inhibitors the steady state plasma concentrations were used to investigate the effect of BBB P-gp inhibition on morphine brain_ECF_ PK. We calculated plasma steady-state (Cp_ss_) concentrations following IV administration (Eq. 1), using published values for plasma clearance (CL), the maintenance or average clinical dose (D), the advised dosing frequency/time interval (τ) and fraction unbound of inhibitor in plasma (fu, p_inhibitor_) collected from the FDA drug label information and/or from literature (Supplementary table 1).

1 $$\:{Cp}_{ss}=\:\frac{D}{CL*\:{\uptau\:}\:}$$

### P-gp inhibitory constants

For the P-gp inhibitors, the plasma steady state concentration and the inhibitory constants K_i_ and/or IC_50_ values were needed. The K_i_ is the inhibitory constant reflecting the binding affinity of an inhibitor to the target (receptor/transporter) while IC_50_ is the concentration required to reduce the target activity to half of the uninhibited condition. The K_i_ and/or IC_50_ were searched via ChEMBL, selecting P-gp (ChEMBL ID: CHEMBL4302), for homo sapiens [27]. Of the selected compounds, the original literature referred to in ChEMBL, was screened and included in the inhibitor database (Supplementary Table 1). For 7 drugs the K_i_ was collected and used for analysis and for the other 27 the IC_50_.

### Quantifying P-gp inhibitory effect

P-gp inhibition by the inhibitors was assumed to occur at the luminal membrane of the BBB having impact on the efflux clearance by P-gp. To quantify this impact on the efflux clearance, the conventional static/basic model for DDI with the Rowland and Matin Eq. 2 was used [28, 29].

2 $$\:{CLe}_{DDI}=\frac{CLe}{(1+\:\frac{{fu,p}_{inhibitor}*{C}_{inhibitor}\:}{Ki})}$$

Here, CLe_DDI_ is the efflux clearance in presence of the inhibitor, CLe is the efflux clearance without inhibitor present, fu, p_inhibitor_ is the unbound fraction in plasma, C_inhbitor_ is the plasma concentration of the inhibitor, and K_i_ is the inhibition rate constant of the inhibitor. In this study, the inhibition is assumed to be reversible, which allows using IC_50_ as a substitute for K_i_ [3]. The CLe_DDI_ needs to be implemented in the active transport mechanism of the LeiCNS-PK3.0 model.

**Fig. 1:**
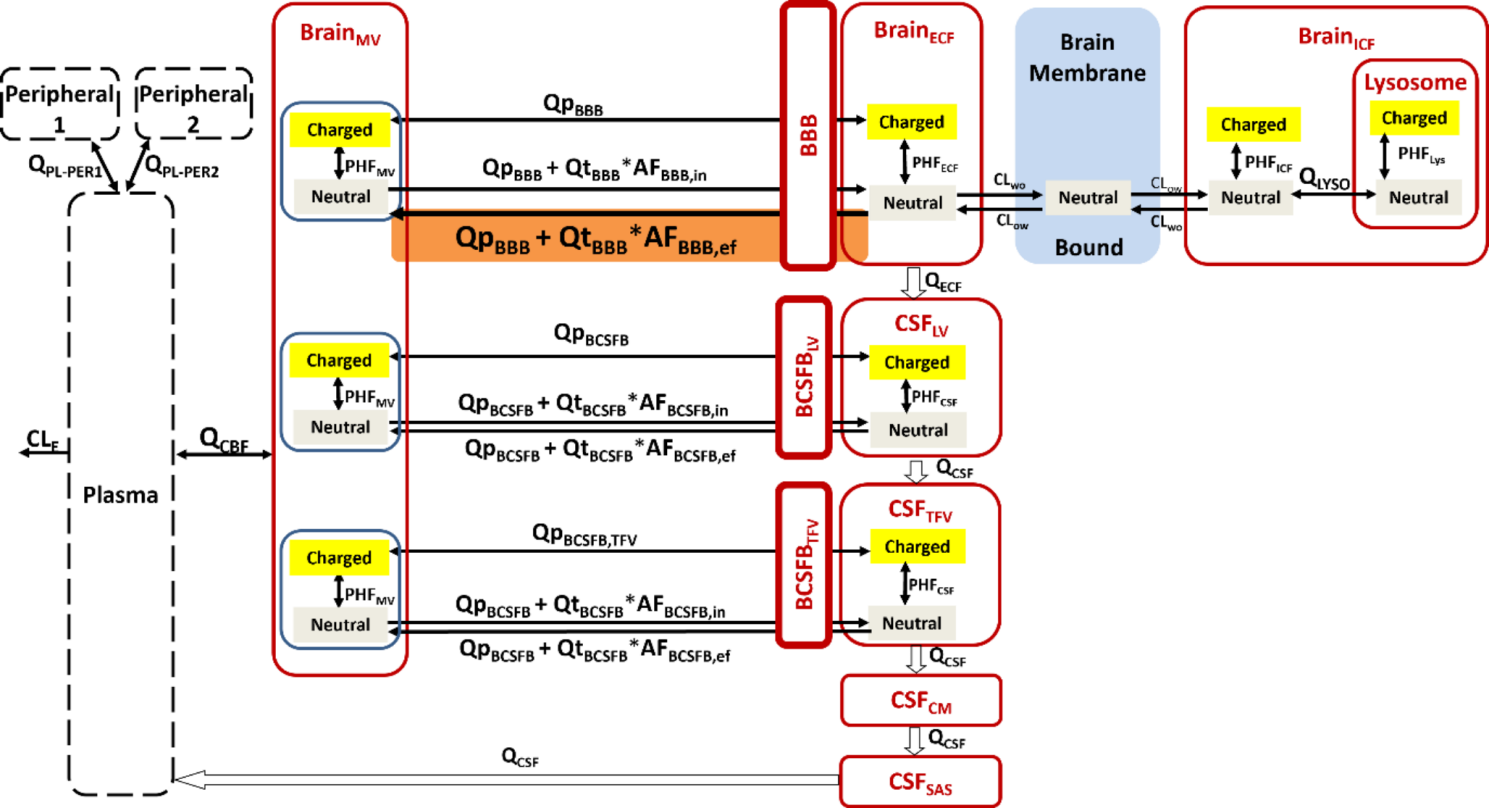
The LeiCNS-PK3.0 model Schematic overview of the central nervous system (CNS) physiologically based pharmacokinetic, the LeiCNS-PK3.0, model including the various CNS and cerebrospinal fluid (CSF) compartments. The orange box indicates the transport route where efflux transporter mediated drug-drug interaction is implemented. Barriers, BBB: blood-brain barrier, BCSFB: blood CSF barrier. Compartments, CM: Cisterna Magna, CSF: cerebrospinal fluid, ECF brain extracellular fluid, LV: lateral ventricles, MV: brain microvasculature, PL: plasma, PER1/2: peripheral compartment 1/2, SAS: subarachnoid space, TFV:3rd, and 4th ventricles. Transport, eff: efflux and in: influx. Flows, CL_E_: central clearance, CL_ow_: lipid-to-water clearance, CL_wo_: water-to-lipid clearance, QCBF: cerebral blood flow, QECF: ECF bulk flow, Q_CSF_: CSF flow, Q_LYSO_: transmembrane clearance of lysosomes, Q_p_: paracellular transport clearance, Q_t_: Transcellular transport clearance. Factors, AF: asymmetry factor, PHF: PH factor

In the LeiCNS-PK3.0 model (Fig. 1), BBB transport is described by passive paracellular (Qp_BBB_), transcellular (Qt_BBB_) and active transport represented by asymmetry factors influx and efflux embedded with the K_p, uu, BBB_ (AFi_BBB_ and AFe_BBB_, respectively). The K_p, uu, BBB_ value indicates the dominance of the transport route across the BBB of the drug with a value above 1 indicating mainly active influx, value around 1 mainly passive transport and a value lower than 1 indicating mainly active efflux. Active transport is embedded in the Qt_BBB_, and here the DDI_effect_ (Rowland and Matin equation as in Eq. 3) was implemented in the Qt_BBB_ in the following equations:

3 $$\:{QtBBB}_{DDI}\:=\frac{QtBBB*AFeBBB}{{DDI}_{effect}}$$

4 $$\:{QtBBB}_{DDI}=\frac{QtBBB*AFeBBB}{(1+\:\frac{{fu,p}_{inhibitor}*{\left[I\right]}_{inhibitor}}{Ki})}$$

Qt_BBB_ is calculated based on the octanol-water partitioning coefficient and the surface area of the BBB while Qp_BBB_ is determined based on the drug’s molecular weight, the diffusion path length across the tight junctions (width across BBB) where paracellular diffusion can take place between the endothelial cells of the BBB and surface area of the BBB (Supplementary Eqs. 1–2). The AFi_BBB_ and AFe_BBB_ are calculated using the K_p, uu, BBB_, the different flows in the CNS and pH values in the brain microvasculature and brain_ECF_ (Supplementary Eqs. 3–4) [19]. For morphine‘s nonlinear BBB transport, a concentration-dependent K_p, uu, BBB_ function [18], which accounts for passive diffusion, active efflux and saturable influx transport [11] was used (Table 1). It should be noted that the concentration dependent K_p, uu, BBB_ function has been established for a clinically relevant dose range between 0.25 and 150 mg/kg, and associated plasma concentrations of morphine (i.e. 0.24 to 145 ng/ml). Within this plasma concentration range the K_p, uu, BBB_ value reaches below 1. Higher dosing of morphine is unrealistic and with that the K_p, uu, BBB_ function is not applicable for these higher concentrations, and ultimately would approach 0, while saturation of all influx and efflux transporters would lead to a K_p, uu, BBB_ that would equal 1 (i.e. no net direction of BBB transport). Active influx and efflux BBB transport parameters in humans (AFi_BBB_ and AFe_BBB_) were derived by using the scaling factor fAFBBB that represents the ratio in transporter protein expression between rats and humans, (see Supplementary Eqs. 3–4) and as derived in previous work by our group [30]. Regarding the probenecid sensitive and saturable active influx transporters of morphine, we assumed these remained equal between rats and humans. Thus, The fAFBBB value of morphine resulted in the value of 0.22, based on the mean total protein P-gp expression at the human BBB of 4.21 fmol/µg [22–24] compared to 19.28 fmol/µg total protein in rats [25, 26]. To see the possible effect of fAFBBB on morphine brain_ECF_ exposure additional analysis was performed for different fAFBBB values (0.1, 0.3 and 1) with tariquidar as inhibitor and was compared to brain_ECF_ exposure with fAFBBB of 0.22.

### Simulation scenarios

Morphine brain_ECF_ exposure was predicted using the LeiCNS-PK3.0 implemented with and without an inhibitor drug to investigate potential DDI. Clinical dosing regimens for all inhibitor drugs were collected from FDA drug labels, using the typical recommended maintenance doses. Intravenous dosing was selected for morphine administration, to not confound our results with systemic P-gp inhibition effects for oral absorption. The recommended intravenous dose for morphine is 0.1–0.2 mg per kg every 4 h as needed [31]. This leads to an intravenous dosing schedule of 10 mg every 4 h based on an average 70 kg individual. Simulations were conducted for seven days to achieve steady state exposures. Additionally, simulations were performed for 50 mg every 4 h, targeting a K_p, uu, BBB_ around 1, to assess the effect of P-gp inhibition in the population. A total of 2000 subjects was simulated, incorporating inter-individual variability in plasma PK as predicted by the population PK model.

### Evaluation metrics

To evaluate the effect of P-gp inhibition at the BBB, morphine brain_ECF_ exposure for morphine alone was compared with morphine used in combination with the use of an inhibitor, focusing on the AUC for the last 24 h obtained after 7 days of treatment. Specifically, the effect on brain_ECF_ exposure in P-gp inhibition was calculated by the area under the curve (AUC_ECF_) DDI ratio of inhibitor over control (Eq. 5):

5 $$\begin{array}{l}\:\:\:\:AUCECF\:DDI\:ratio\:\\=\:\frac{{AUC}_{ECF,inhibited}}{{AUC}_{ECF,control}}\:*100\%\:\end{array}$$

The AUC_ECF_ ratios are further studied by comparing the fraction in the populations (percentiles) by summarizing the AUC_ECF_ DDI ratios (Eq. 5) in the 95, 97.9, 99 and 99.9 percentiles of the simulated 2000 subjects.

### Sensitivity analysis

A sensitivity analysis was performed on the inhibitory drug parameters C_inhibitor_, fu, p_inhibitor_ and K_i_ or IC_50_, to evaluate the effect of variations of this parameter on the potential P-gp mediated DDI effect at BBB on brain_ECF_ exposure. The absolute values of the three parameters were simulated using fold-change factors of 0.1, 10, 100 and 1000 fold change. Furthermore, to evaluate the potential effect of nonlinear BBB transport, besides the already simulated 10 mg, non-clinical doses of 50 and 100 mg every 4 h were also simulated in the sensitivity analysis also. These different doses would due to nonlinear transport lead to K_p, uu, BBB_ values of above, around and below 1. In addition, sensitivity analysis was performed on the fAFBBB (0.1, 0.22, 0.3 and 1) with various tariquidar concentrations (1, 10 and 100 fold). Furthermore, simulations were conducted for 100 mg of morphine (K_p, uu, BBB_ > 1), with and without active efflux (AFe_BBB_), using loratadine, tariquidar, and verapamil as inhibitors to assess the impact of active efflux transport.

### Software

Simulations for nonlinear BBB transport and LeiCNS-PK3.0 models were performed using the package RxODE version 1.1.5 and for sensitivity analysis the additional PKNCA package version 0.9.5 using R version 4.1.3.

## Results

### P-gp inhibition effect on morphine brainECF exposure

**Fig. 2:**
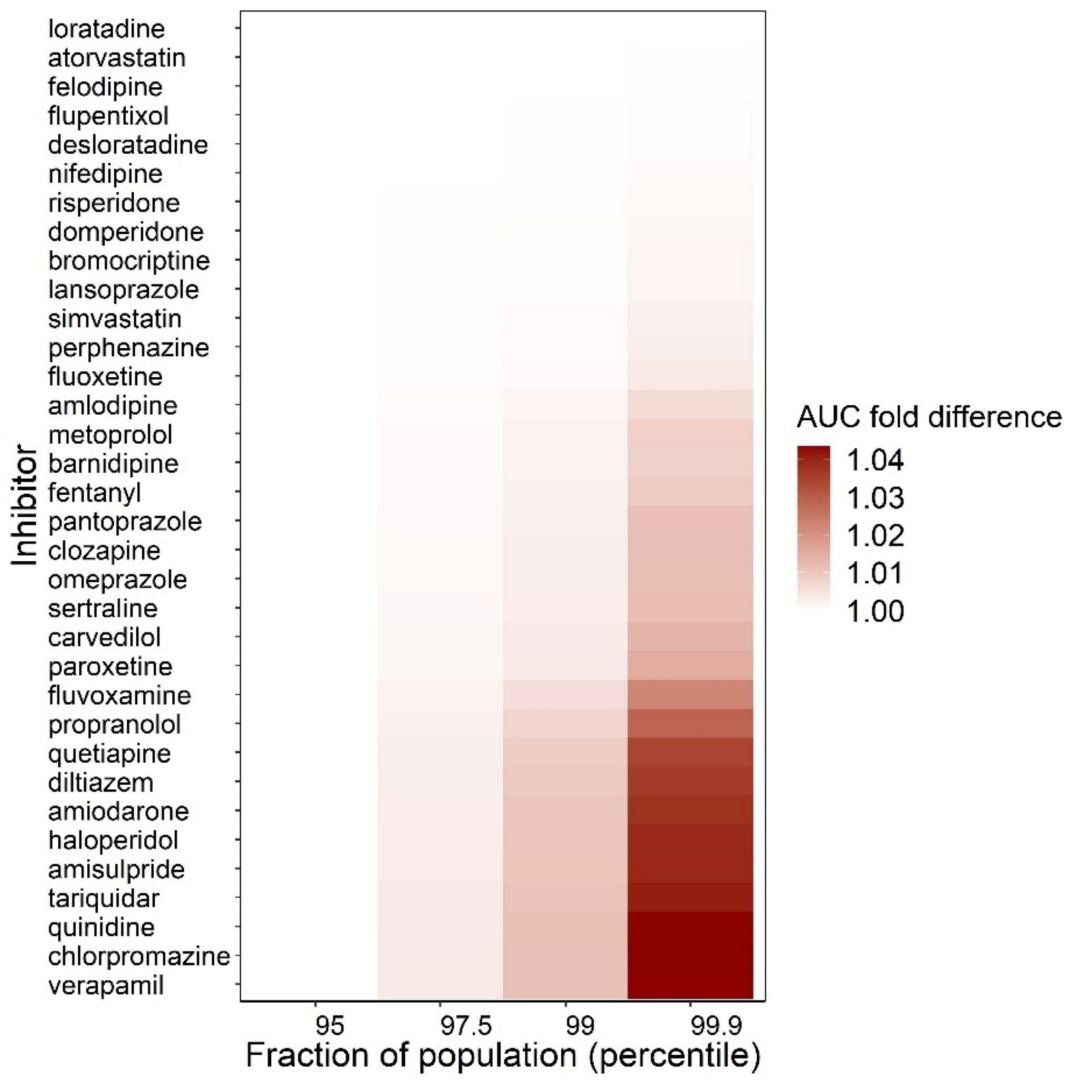
P-gp inhibition effect on morphine brain extracellular fluid (brain_ECF_) exposure ratio in simulated population shown as area under the curve in drug-drug interaction (AUC_ECF_DDI ratio). Morphine brain_ECF_ exposure was compared in the absence and presence of an inhibitor. The results were compared for various percentiles within the simulated population group. The larger the AUC_ECF_ DDI ratio percentiles of the simulated patients in the population, the higher the chance on observing increase in morphine brain_ECF_ in inhibited condition

We simulated morphine administration of 10 mg every 4 h in the absence and presence of an inhibitor. Tariquidar was used as positive control of P-gp inhibition, as tariquidar is a well-known specific, potent, and noncompetitive P-gp inhibitor. As the simulations included inter-individual variability for morphine, we calculated the change in AUC_ECF_ for different percentiles of simulated patients in the population. These analyses demonstrate that for all P-gp inhibitors evaluated, when used at their clinical doses, there was no clinically relevance impact on changes in morphine (Fig. 2). Even when considering the specific AUC_ECF_ DDI ratio percentiles of patients who have the highest morphine brain_ECF_ exposures due to inter-patient variability in PK, only a modest fold change of < 4% was observed.

### Sensitivity analysis

**Fig. 3:**
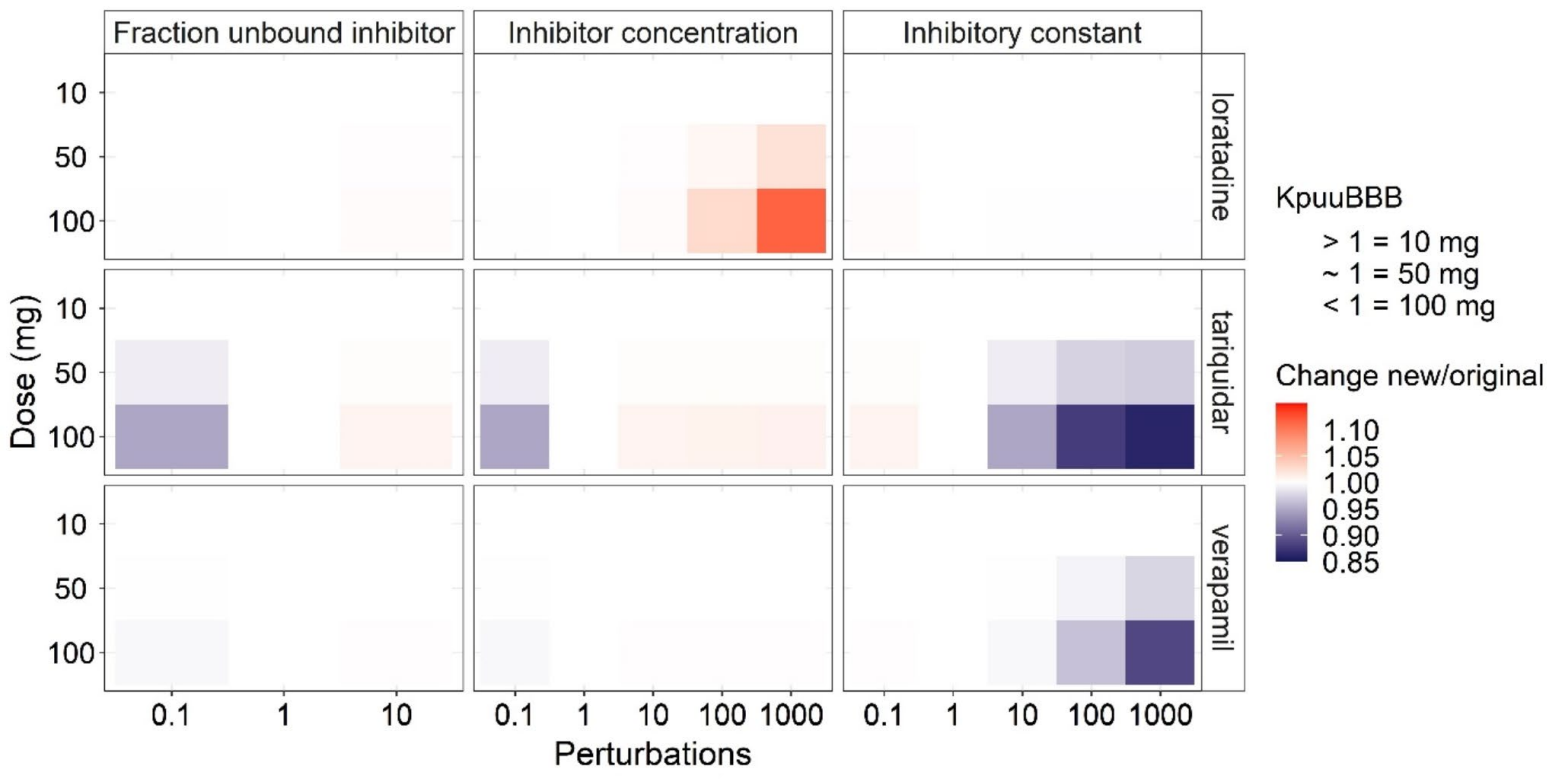
Sensitivity analysis on the inhibitory constants and effect on morphine brain exposure with three P-glycoprotein inhibitors. A sensitivity analysis for morphine 10, 50 and 100 mg 6 times a day administration was simulated under inhibition of loratadine, tariquidar or verapamil at brain extracellular fluid (brain_ECF_). The absolute values of inhibitor constant (Ki or IC_50_) and inhibitor concentration were multiplied by perturbation factors of 0.1, 10, 100 and 1000. The fraction unbound of inhibitor were perturbated with only 0.1 and 10 to keep the absolute value between physiological relevant values of 0 and 1. The morphine brain_ECF_ exposure ratios under the altered conditions were compared to control condition (factor 1). K_p, uu, BBB_ = ratio of extent of drug transporter across the blood brain barrier (BBB) and indicated the main route of transport across BBB. A K_p, uu, BBB_ of < 1 indicates dominance of influx transporters, ~ 1 indicates passive transport and > 1 indicates dominance of efflux transporters

Previous research has suggested a low plasma concentration of an inhibitor as main reason for the minor inhibition by P-gp at BBB [6]. Therefore, a sensitivity analysis was performed on all the inhibitor parameters were C_inhbitor_ and K_i_ or IC_50_, were altered by 0.1-, 10-, 100- and 1000-fold and fu, p_inhibitor_ by 0.1 and 10 to remain the absolute value between physiological relevant values of 0 and 1 for the weakest inhibitor loratadine, strongest inhibitor verapamil and positive control tariquidar. To assess the impact of nonlinear BBB transport of morphine, different doses of 10, 50 and 100 mg of morphine 6 times a day was simulated, whereby every dose results in a K_p, uu, BBB_ value of above 1, around 1 and lower 1, respectively due to nonlinear transport. In general, we find that the DDI effect for all three inhibitors on morphine brain_ECF_ PK remains minimal (Fig. 3). Minor effects are observed at higher doses of morphine, where the impact of its BBB influx transport diminishes by getting saturated and efflux transport dominates. For 100 mg of morphine whereby the K_p, uu, BBB_ value will be lower than 1, reduction of either the fu, p_inhibitor_ or the plasma C_inhbitor_ would result in lowering of morphine brain_ECF_ exposure up to 12%, while increasing these two parameters separately could lead to an increase of morphine brain_ECF_ exposure up to 3%. The impact of changing the inhibitory constant (K_i_ or IC_50_) was predicted to be the opposite of fu, p_inhibitor_ and C_inhbitor_ lower constant resulted in less than 1% increase in morphine brain_ECF_ exposure while increasing the constant resulted in up to 13% decrease. At a perturbation of 100-fold for the inhibitory constant we have seen for verapamil which is a stronger inhibitor less effect compared to tariquidar which could be explained because of the Ki of verapamil is smaller than (fu, p_inhibitor_*C_inhibitor_) even when the K_i_ is increased with 100-fold, while once tariquidar K_i_ is increased 100-fold the K_i_ becomes larger than (fu, p_inhibitor_*C_inhibitor_). When the K_i_ is increased a 1000-fold, also for verapamil an increased effect is observed.

Furthermore, the additional analysis on K_p, uu, BBB_ demonstrates how at higher morphine doses larger differences in the brain_ECF_ exposure are expected (Supplementary figure S1). Also analysis at a dose of 100 mg 6 times per day administration of morphine with and without efflux transport (AFe_BBB_ = 1) the maximum effect of P-gp inhibition can be observed (Supplementary figure S2).

### Impact translational factor on morphine brainECF exposure

**Fig. 4:**
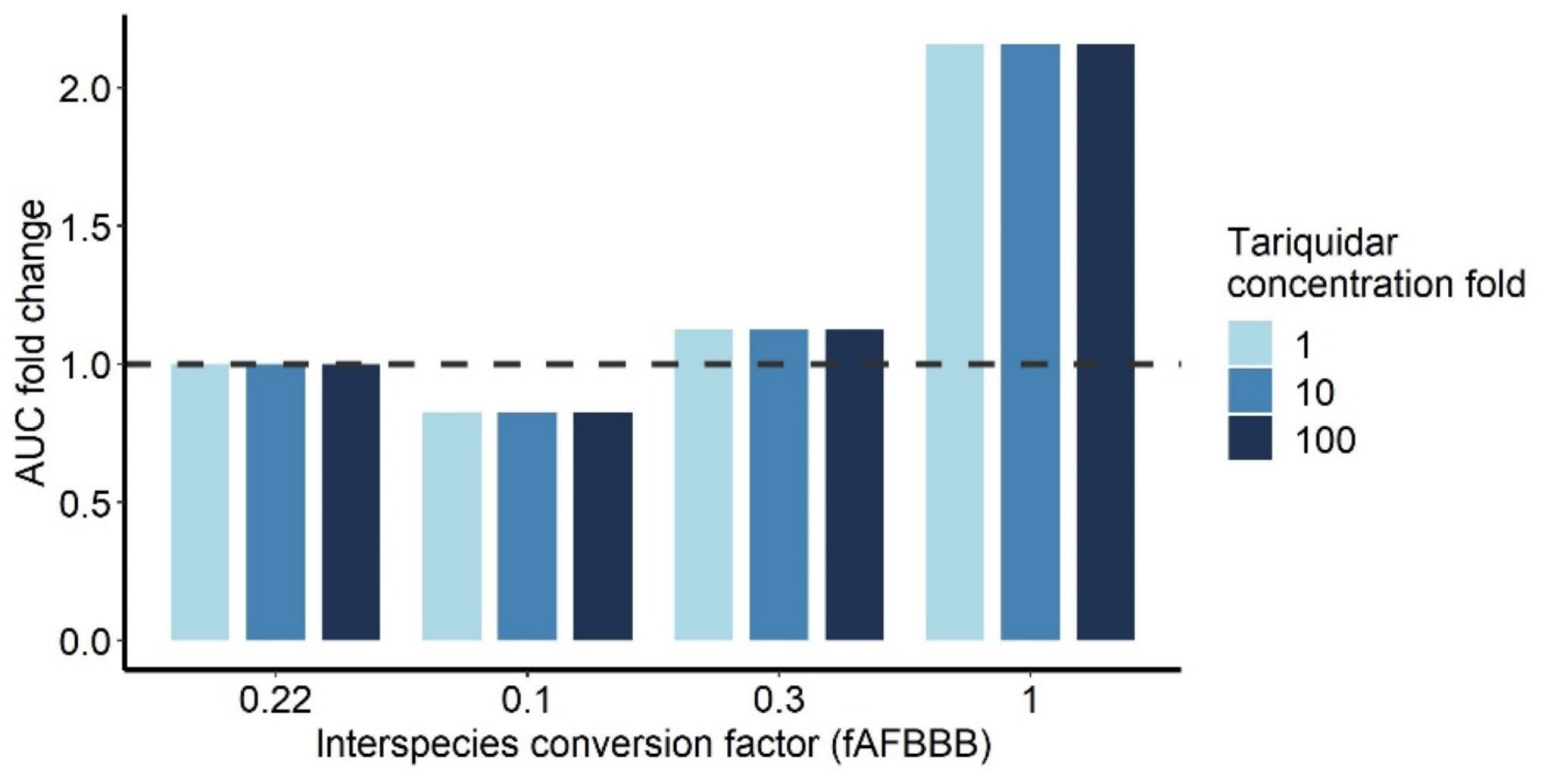
Morphine brain extracellular fluid (brain_ECF_) under the curve (AUC) fold change in the absence and presence of P-glycoprotein inhibitor with different values for the translational factor. The translational factor, fAFBBB, is perturbated to different values to see the effect on morphine brain_ECF_ AUC for 10 mg of 6 times a day administration. The dashed line represents the control condition with no P-gp inhibitor present. The different bar colors represent the different concentrations of P-gp inhibitor, tariquidar

Morphine simulations were conducted using different fAFBBB values and various concentrations of tariquidar to assess their impact on morphine brain_ECF_ exposure. The analyses demonstrated that changing the fAFBBB value alters the relative morphine brain_ECF_ AUC, with higher fAFBBB values resulting in increased morphine brain_ECF_ AUC, as shown in Fig. 4. Additionally, the results indicated that the inhibition caused by increasing tariquidar concentrations remained consistent across different fAFBBB values.

## Discussion

In this study the impact of P-gp mediated DDI at the level of the BBB on morphine brain_ECF_ exposure was explored using the LeiCNS-PK3.0 PBPK model framework. Our analysis shows that many commonly used clinical P-gp inhibitor drugs do not have a significant effect on morphine brain_ECF_ exposure as shown for several clinically relevant dosing regimens of morphine.

The negligible DDI effects on CNS exposure observed in our study is in line with previously published research. Earlier it has been shown that intravenous and oral morphine administration in combination with oral quinidine or intravenous PSC833 as inhibitor resulted in minimal changes in systemic exposure and CNS activity in humans [32–34]. Similar results were observed for other opioid P-gp substrates fentanyl and methadone [35, 36]. Clinical studies using PET imaging have shown inhibition of P-gp up to 75% once verapamil and loperamide with tariquidar, cyclosporin A or quinidine was administered [6, 8, 9, 37–39]. One of the findings of these studies was that the distribution volume or brain uptake of loperamide by tariquidar could increase up to 4-fold once the administered tariquidar doses exceeded the clinically recommended dose [39]. It should be noted that loperamide is a stronger P-gp substrate than morphine, and thereby more sensitive to P-gp efflux than morphine [40, 41] and therefore the sensitivity of loperamide for P-gp mediated DDI is expected to be larger. Noteworthy is that PET reflects total brain concentrations (actually ligand activity, that may also reside on metabolites) and the observations could lead to overestimation of BBB extent because it shows the unbound and bound concentrations. To convert the PET measured total concentrations to unbound concentrations, a correction by brain tissue binding and brain intracellular distribution represented by parameter the unbound volume of distribution volume (V_u, brain_) can be done [42]. Once the PET brain concentrations are corrected to unbound brain_ECF_ concentrations, the extent at BBB will be lower and possible observed P-gp inhibition effect can be lower. This would be in line with our study results and suggest that this approach may be used to study DDIs for other drug interactions, after further validation.

Increasing the plasma concentrations of a P-gp inhibitor resulted in a negligible increase in morphine exposure at brain_ECF_. This even holds true for higher doses of morphine whereby the BBB influx transport of morphine is saturated and efflux transport dominates the extent of morphine’s BBB transport. By comparing the plasma unbound maximum concentration of the inhibitors to its K_i_ or IC_50_ value, Kalvass et al. (2013) suggested that the values were equivalent to or lower for which the inhibition would be less than 2-fold [6]. Our sensitivity analysis of inhibitor concentration do not support previous literature, which posited that the relatively low clinical P-gp inhibition is primarily due to low systemic unbound concentrations of the inhibitor [6].

Changes in the inhibitory constant, K_i_ or IC_50_, can also have an impact on morphine brain_ECF_ exposure. The K_i_ or IC_50_ values used in this study are derived from distinct cell lines that have been transfected with the human P-gp (*MDR*1) gene. This may yield varying levels of P-gp expression across diverse cell lines. This was shown by Kuteykin-Teplyakov et al. (2010), who found that the relative expression of human Mdr1 mRNA (P-gp) was relatively higher in LLC cells compared to MDCK-II [43]. Different relative P-gp expression can influence the extent of P-gp inhibition. In addition, the experimental determination of the inhibitory constant leads to some uncertainty in its exact value. However, our sensitivity analysis indicates that even in case of a 100-fold difference in Ki, the impact on morphine brain_ECF_ exposure would remain minimal. It is also noteworthy to acknowledge that not all cell lines accurately reflects either the BBB or the potential functionality of P-gp at the BBB. In this study, P-gp inhibition is presumed to be contingent on the drug and independent of cell line specificity. Based on the sensitivity analysis results, the impact of altered inhibitory constant would have a minor impact on morphine BBB transport.

Although this study is focused on P-gp mediated DDI effects, morphine is also substrate for other transporters at the BBB (i.e. probenecid sensitive efflux transporters and the saturable influx transporter) [10–14]. These additional transporters might have an impact on our predictions in two ways, the translational factor fAFBBB and DDI arise from interaction on multiple transporters. Firstly, alterations in fAFBBB do solely impact the relative morphine brain_ECF_ exposure and do not affect the DDI. The impact of varying fold changes in tariquidar concentrations on brain_ECF_ AUC shows that these concentration do not affect morphine brain_ECF_ AUC. However, the brain_ECF_ AUC fold change increases as fAFBBB values rise. This because the AF factors are corrected with the fAFBBB, and higher fAFBBB values lead to larger AF factors, which in turn increases the morphine brain_ECF_ AUC. Morphine brain_ECF_ exposures are driven by multiple transport processes. At present, only P-gp has been specifically scaled for expression and its nonlinear effects on morphine concentrations have been incorporated. Other active transport processes are currently grouped with P-gp and assumed to scale uniformly across species. While this assumption may influence morphine brain_ECF_ exposure, it is not expected to have a direct effect on the DDI. Secondly, transporter mediated DDI could also occur by inhibition of probenecid sensitive transporter or induction of the influx transporter or an synergistic effect of dual inhibition. Synergistic effect have been previously shown for lapatinib that inhibition of P-gp and breast cancer resistant protein (BCRP) resulted in a disproportional increase in lapatinib CNS exposure [44]. These additional transporter inhibition could possible alter the morphine brain_ECF_ exposure and therefore should be studied further.

Outside the scope of this study, to have a broad overview of the P-gp inhibition at multiple body sites, the P-gp inhibition at gastrointestinal level could be taken into consideration. Increase of morphine brain_ECF_ exposure could be simulated by including the P-gp inhibition at the gastrointestinal level (potentially leading to changes in plasma exposure). Earlier, a clinical study focusing on the intestinal P-gp inhibition effect on oral morphine by oral quinidine compared to placebo, showed significant increase in morphine peak concentrations and plasma AUC [32]. Many other possibilities aiming to inhibit gastrointestinal P-gp inhibition is being studied to increase drug absorption and bioavailability given in overviews by various reviews [45, 46]. Incorporation of active transport processes at the BBB such as described in the current model would be relevant to incorporate also in PBPK models and software platforms, which already have incorporated P-gp effects at the gastrointestinal tract.

We have described a CNS PBPK modeling approach for prediction of DDIs at the level of the BBB and associated changes in CNS drug distribution. We find that P-gp inhibition at the BBB does not result in clinically relevant changes in morphine brain_ECF_ exposure. These findings are in line with the relatively modest overall contribution of P-gp-mediated BBB transport of morphine. However, for other CNS-targeting drugs which are stronger substrates, more pronounced effects can be expected. In this context, the framework implemented in this study can be of relevance to support the systematic assessment of mediated DDIs at the BBB for other drugs, and potentially other transporters present at the BBB [3, 4].

## Electronic supplementary material

Below is the link to the electronic supplementary material.

Supplementary Material 1

## Data Availability

No datasets were generated or analysed during the current study.
