# Supplementary material for: Do P-glycoprotein-mediated drug-drug interactions at the blood-brain barrier impact morphine brain distribution?
Source: J Pharmacokinet Pharmacodyn. 2025 Jan 7;52(1):11. doi: 10.1007/s10928-024-09957-0 (PMC11706904; doi:10.1007/s10928-024-09957-0)
Supplement: Supplementary file 1 — Supplementary Material 1 [file 10928_2024_9957_MOESM1_ESM.docx]

**Do P-glycoprotein-mediated drug-drug interactions at the blood-brain barrier impact morphine brain distribution?**

Berfin Gülave^1^, Ariel Lesmana^2^, Elizabeth CM de Lange^3^, JG Coen van Hasselt^4^

1. Division of Systems Pharmacology and Pharmacy, Leiden Academic Center for Drug Research, Leiden University, Leiden, The Netherlands. ORCID ID: 0000-0001-8954-882X
2. Division of Systems Pharmacology and Pharmacy, Leiden Academic Center for Drug Research, Leiden University, Leiden, The Netherlands.
3. Division of Systems Pharmacology and Pharmacy, Leiden Academic Center for Drug Research, Leiden University, Leiden, The Netherlands. ORCID ID: 0000-0001-8303-1117
4. Division of Systems Pharmacology and Pharmacy, Leiden Academic Center for Drug Research, Leiden University, Leiden, The Netherlands. ORCID ID: 0000-0002-1664-7314

Correspondence to email: coen.vanhasselt@lacdr.leidenuniv.nl, telephone: +31 71 527 3266, address: Leiden Academic Centre for Drug Research, Leiden University, Einsteinweg 55, 2333 CC, Leiden, The Netherlands

**Supplementary table 1. P-glycoprotein inhibitor with their inhibitor coefficients value and plasma pharmacokinetics**

|  |  |  |  |  |  | **Plasma pharmacokinetics** | | | |  |
| --- | --- | --- | --- | --- | --- | --- | --- | --- | --- | --- |
| **Drug** | **Parameter** | **Value (µM) [range]** | **Cell line** | **Reference** | **fu,p** | **Clearance (ml/min)** | **Dose (mg)** | **Interval (hr)** | **C_ss_ (ng/ml)** | **DDI**** |
| amiodarone | Ki | 5.78 | L-MDR1 | [1, 2] | 0.04 | 385 | 400 | 24 | 721.5 | 7.7 |
| amisulpride | IC50 | 123.5 | Caco-2 | [3] | 0.725 | 401.67 | 400 | 24 | 691.6 | 11.0 |
| amlodipine | IC50 | 22 | LLC-GA5-COL150 | [4] | 0.02 | 42* | 5 | 24 | 82.7 | 0.2 |
| atorvastatin | IC50 | 289 [271-307] | 3T3-G185/CF1R12 | [5] | 0.02 | 625 | 40 | 24 | 44.4 | 0.01 |
| barnidipine | IC50 | 10.6 [8.6-12.6] | LLC-GA5-COL150 | [4, 6] | 0.08 | 379 | 10 | 24 | 18.3 | 0.3 |
| bromocriptine | IC50 | 3.39 [2.81-3.96] | L-MDR1 | [2] | 0.08 | 5180* | 10 | 24 | 1.3 | 0.05 |
| carvedilol | IC50 | 5.7 [4.6-6.6] | NIH-3T3-G185 | [7] | 0.02 | 600 | 25 | 12 | 57.9 | 0.5 |
| chlorpromazine | Ki | 0.6 | NA | [8] | 0.05 | 593 | 300 | 8 | 1054 | 275.5 |
| clozapine | IC50 | 92.78 [92.78- >100] | L-MDR1/Caco-2 | [3, 9] | 0.03 | 505 | 150 | 12 | 412.5 | 0.4 |
| desloratadine | IC50 | 43 | NIH-3T3-G185 | [10] | 0.15 | 1995* | 5 | 24 | 1.7 | 0.02 |
| diltiazem | IC50 | 56.4 [42.66-77.7] | LLC-GA5-COL150/A2780adr | [6, 11, 12] | 0.25 | 600 | 240 | 12 | 555.6 | 5.9 |
| domperidone | IC50 | 45.64 | Caco-2 | [3] | 0.08 | 625 | 10 | 24 | 11.1 | 0.05 |
| felodipine | IC50 | 29.3 [26.3 - >60] | NIH-3T3-G185 | [7] | 0.01 | 800 | 10 | 24 | 8.7 | 0.01 |
| fentanyl | IC50 | 6.5 | Caco-2 | [13] | 0.175 | 583.3* | 0.8 | 6 | 3.8 | 0.3 |
| fluoxetine | IC50 | 115.5 | L-MDR1 | [14] | 0.055 | 604.8* | 60 | 24 | 68.9 | 0.1 |
| flupentixol | IC50 | 18.32 | Caco-2 | [3] | 0.01 | 290 | 5 | 24 | 12 | 0.02 |
| fluvoxamine | IC50 | 146.7 | Caco-2 | [3] | 0.2 | 490.25 | 100 | 12 | 283.3 | 1.2 |
| haloperidol | Ki | 0.2 | NA | [8] | 0.1 | 910* | 5 | 12 | 7.6 | 10.2 |
| lansoprazole | IC50 | 62.8 | Caco-2 | [15] | 0.03 | 525 | 30 | 24 | 39.7 | 0.05 |
| loratadine | IC50 | 11.4 | NIH-3T3-G185 | [10] | 0.02 | 14000* | 10 | 24 | 0.5 | 0.00 |
| metoprolol | Ki | 200 | NA | [8] | 0.1 | 1000 | 200 | 24 | 138.9 | 0.3 |
| nifedipine | IC50 | 472 | LLC-GA5-COL150 | [6] | 0.05 | 575 | 60 | 24 | 72.5 | 0.02 |
| omeprazole | IC50 | 17.7 | Caco-2 | [15] | 0.05 | 550 | 40 | 24 | 50.5 | 0.4 |
| pantoprazole | IC50 | 17.9 | Caco-2 | [15] | 0.02 | 157.5 | 30 | 24 | 132.3 | 0.4 |
| paroxetine | IC50 | 29.8 | L-MDR1 | [14] | 0.05 | 300 | 50 | 24 | 115.7 | 0.6 |
| perphenazine | IC50 | 15.45 | Caco-2 | [3] | 0.05 | 1666.67 | 8 | 8 | 10 | 0.08 |
| propranolol | Ki | 48 | NA | [8] | 0.1 | 810 | 160 | 12 | 274.3 | 2.2 |
| quetiapine | IC50 | 12.8 [0.8-24.8] | L-MDR1/Caco2 | [3, 9] | 0.17 | 1666.67 | 150 | 12 | 125 | 4.3 |
| quinidine | Ki | 12 | NA | [8] | 0.14 | 280* | 400 | 6 | 3968.2 | 142.7 |
| risperidone | IC50 | 25.9 [5.87-63.26] | Caco-2/LLC-PKI/L-MDR1 | [9, 16] | 0.1 | 833.33 | 4 | 24 | 3.3 | 0.03 |
| sertraline | IC50 | 31.8 | L-MDR1 | [14] | 0.02 | 614.67* | 200 | 24 | 216.4 | 0.4 |
| simvastatin | IC50 | 30.6 [8.9-56.8] | NIH-3T3-G185 | [5, 7] | 0.05 | 1470.83 | 40 | 24 | 18.9 | 0.07 |
| tariquidar | IC50 | 0.23 [0.125-0.33] | A2780adr | [12, 17] | 0.005 | 208 | 150 | 24 | 500.8 | 16.8 |
| verapamil | Ki | 0.3 | NA | [8] | 0.1 | 502 | 120 | 8 | 498 | 365.1 |

* Calculated for a 70kg person

**DDI = (fu,p * Css) / Ki or IC50

CNS = central nervous system

Ki = inhibition rate constant describing inhibitory affinity of the inhibitor

IC50 = concentration of inhibitor needed to reduce 50% of transporter activity

fu,p = fraction unbound in plasma

cell lines (all transfected with human *MDR1* gene encoding P-gp):

- L-MDR1 & LLC-PK1 = porcine kidney epithelial cell line
- Caco-2 = colorectal adenocarcinoma cell line
- LLC-GA5-COL150 = LLC-PK1 cell line incubated in medium with 150ng/ml colchicine
- NIH-3T3-G185 = mouse fibroblast cell line
- A2780adr = adriamycin-resistant cell line

**Supplementary equations**

The following equations have been used to calculate different modes of transport across the blood-brain barrier (BBB) and the asymmetry factors (AFs) [18].

Paracellular clearance across the BBB (Qp_BBB_) was calculated using the aqueous diffusivity of the drug (Daq, cm^2^/sec), the molecular weight of the drug (MW, in g/mol), the diffusion width across BBB (width_BBB_), and the BBB surface area (SAp_BBB_) of BBB. The BBB surface area was corrected with a factor that represent the surface area of BBB where the effective paracellular transport takes place.

**Eq 1** ${Qp}_{BBB}=\frac{Daq}{{width}_{BBB}}*{SAp}_{BBB}$

Transcellular clearance across the BBB (QtBBB) was calculated based on the transmembrane permeability of the drug (P0transcellular, cm/sec), the octanol-water partition coefficient (logP) of the drug, and the surface area (SAtBBB) of the BBB. The BBB surface area was corrected with an effective surface area factor for BBB to account for surface area dedicated for transcellular transport only.

**Eq 2** ${Qt}_{BBB}= 0.5* P_{0}^{transcellular}*{SAt}_{BBB}$

Equations calculating the influx and efflux AFs at the BBB are based on the K­_p,uu,BBB_ value:

- If K_p,uu,BBB_ = 1 then both AFi_BBB_  and AFe_BBB_ are set to 1 {AFi_BBB_ = 1; AFe_BBB_ = 1}

- If K_p,uu,BBB_ < 1 then AFe_BBB_ is set to 1 and AFi_BBB_ calculated according to **Eq 3** {AFi_BBB_ =**Eq 3**; AFe_BBB_ = 1}

- If K_p,uu,BBB_ < 1 then AFi_BBB_ is set to 1 and AFe_BBB_ calculated according to **Eq 4** {AFi_BBB_ = 1; AFe_BBB_ = **Eq 4**}

**Eq 3** ${AFi}_{BBB}=-\frac{K_{p,uu,BBB}*Q_{CBF}*Q_{ECF}+{K_{p,uu, CM}*Qp}_{BBB}*Q_{CSF}+Q_{CBF}*(K_{p,uu, BBB}*({AFe}_{BBB}*{PHF}_{ECF}*{Qt}_{BBB}+{Qp}_{BBB})-{Qp}_{BBB})}{{PHF}_{MV}*{Qt}_{BBB}*(K_{p,uu,CM}*Q_{CSF}-Q_{CBF})}$

**Eq 4** ${AFe}_{BBB}=-\frac{K_{p,uu,BBB}*Q_{CBF}*Q_{ECF}+K_{p,uu, CM}*Q_{CSF}*({AFi}_{BBB}*{PHF}_{MV}*{Qt}_{BBB}+{Qp}_{BBB})+Q_{CBF}*(K_{p,uu,BBB}*{Qp}_{BBB}-{AFi}_{BBB}*{PHF}_{MV}*{Qt}_{BBB}-{Qp}_{BBB})}{K_{p,uu, BBB}*{PHF}_{ECF}*{Qt}_{BBB}*Q_{CBF}}$

Supplementary equations notations

AFe_BBB_: efflux asymmetry factor across the blood brain barrier

AFi_BBB_: influx asymmetry factor across the blood brain barrier

K_p,uu,BBB_: brain extracellular fluid-to-plasma unbound drug concentration ratio

K_p,uu,CM_: cisterna magna-to-plasma unbound drug concentration ratio

PHF_ECF_: pH factor of brain extracellular fluid

PHF_MV_: pH factor of brain microvasculature

Q_CBF_: cerebral blood flow

Q_CSF_: cerebrospinal fluid flow

Q_ECF_: brain extracellular fluid bulk flow

Qp_BBB_: paracellular transport clearance at blood brain barrier

Qt_BBB_: transcellular transport clearance at blood brain barrier


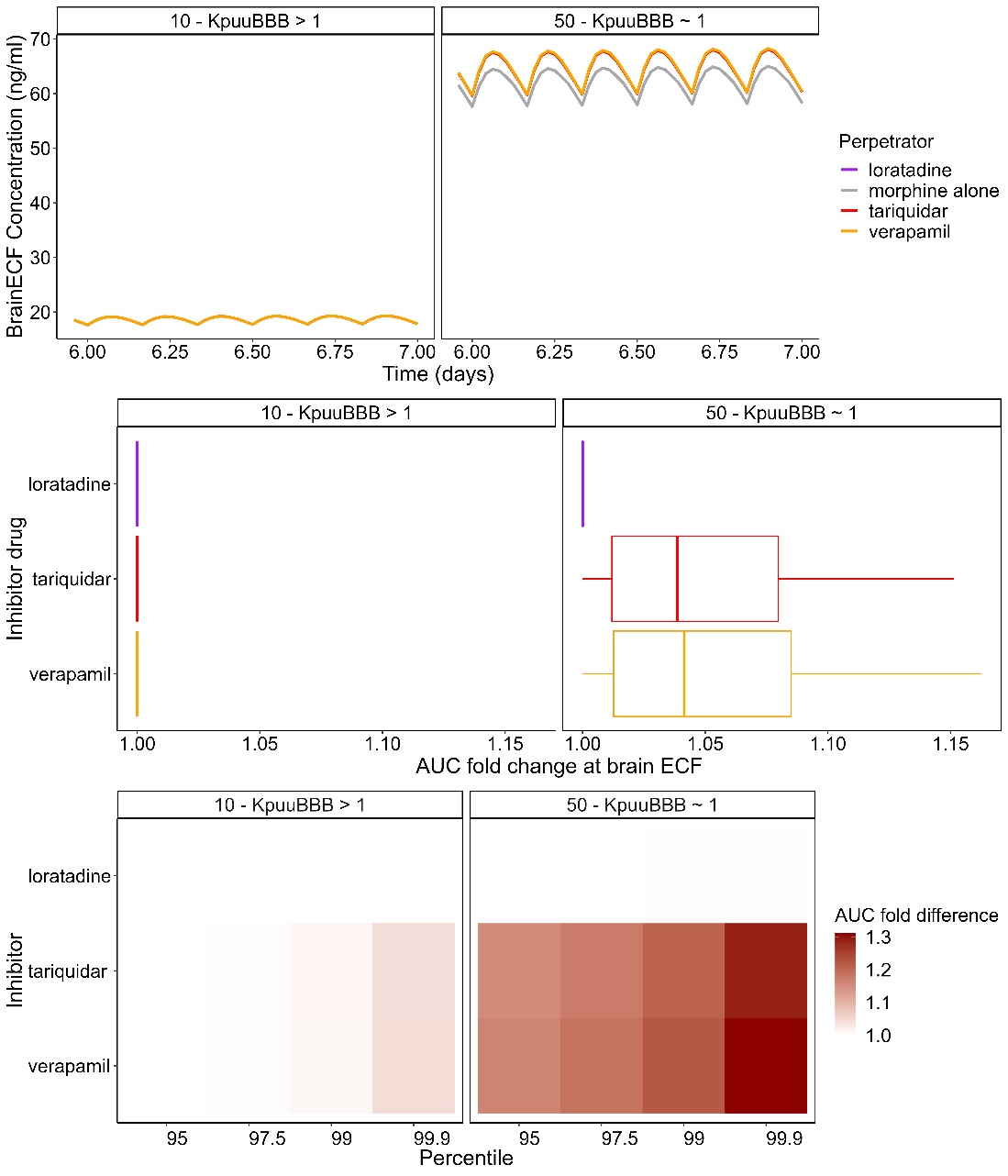


**Figure S1. P-gp inhibition effects for three P-gp inhibitors on the change in morphine brain extracellular fluid drug exposure (brain_ECF_ AUC) ratio in a simulated patient population.** Morphine brain_ECF_ exposure was compared in the absence and presence of an inhibitor at a dose of 10 mg and 50 mg 6 times a day. The results were compared for various AUC exposure percentiles within the simulated population group.


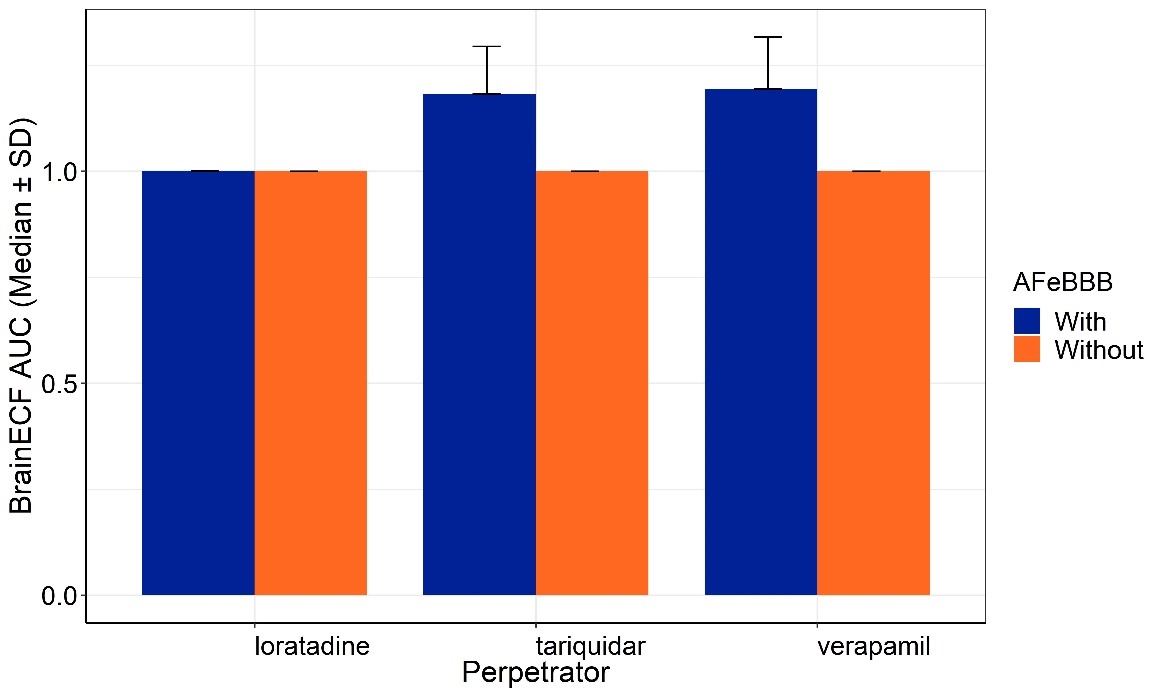


**Figure S2. Morphine brain extracellular fluid (brain_ECF_) exposure under inhibition by 3 P-glycoprotein inhibitors with and without active efflux (AFe_BBB_).** Morphine exposure shown are area under the curve (AUC) under inhibited over control condition in condition with (AFe_BBB_ is calculated according **Eq 4**) and without active efflux (AFe_BBB_ is set to 1) at a dose of 100mg 6 times a day administration. The results are shown as median AUC change and standard deviation (SD).

**References**

1. Yasuda K, Lan L Bin, Sanglard D, Furuya K, Schuetz JD, Schuetz EG (2002) Interaction of cytochrome P450 3A inhibitors with P-glycoprotein. J Pharmacol Exp Ther 303:323–332. https://doi.org/10.1124/jpet.102.037549

2. Ekins S, Kim RB, Leake BF, Dantzig AH, Schuetz EG, Lan L, Yasuda K, Shepard RL, Winter MA, Schuetz JD, Wikel JH, Wrighton SA, Lilly E, Indiana SE (2002) Three-Dimensional Quantitative Structure-Activity Relationships of Inhibitors of P-Glycoprotein. Mol Pharmacol 61:964–973. https://doi.org/10.1124/mol.61.5.964

3. Abou El Ela A, Härtter S, Schmitt U, Hiemke C, Spahn-Langguth H, Langguth P (2010) Identification of P-glycoprotein substrates and inhibitors among psychoactive compounds — implications for pharmacokinetics of selected substrates. J Pharm Pharmacol 56:967–975. https://doi.org/10.1211/0022357043969

4. Katoh M, Nakajima M, Yamazaki H, Yokoi T (2000) Inhibitory potencies of 1,4-dihydropyridine calcium antagonists to P-glycoprotein-mediated transport: Comparison with the effects on CYP3A4. Pharm Res 17:1189–1197. https://doi.org/10.1023/A:1007568811691

5. Wang EJ, Casciano CN, Clement RP, Johnson WW (2001) HMG-CoA reductase inhibitors (statins) characterized as direct inhibitors of P-glycoprotein. Pharm Res 18:800–806. https://doi.org/10.1023/A:1011036428972

6. Takara K, Sakaeda T, Tanigawara Y, Nishiguchi K, Ohmoto N, Horinouchi M, Komada F, Ohnishi N, Yokoyama T, Okumura K (2002) Effects of 12 Ca2+ antagonists on multidrug resistance, MDR1-mediated transport and MDR1 mRNA expression. Eur J Pharm Sci 16:159–165. https://doi.org/10.1016/S0928-0987(02)00082-9

7. Wang E jia, Casciano CN, Clement RP, Johnson WW (2001) Active transport of fluorescent P-glycoprotein substrates: Evaluation as markers and interaction with inhibitors. Biochem Biophys Res Commun 289:580–585. https://doi.org/10.1006/bbrc.2001.6000

8. Pajeva IK, Wiese M (2002) Pharmacophore Model of Drugs Involved in P-Glycoprotein Multidrug Resistance : Explanation of Structural Variety ( Hypothesis ). J Med Chem 45:5671–5686

9. Wang JS, Zhu HJ, Markowitz JS, Donovan JL, DeVane CL (2006) Evaluation of antipsychotic drugs as inhibitors of multidrug resistance transporter P-glycoprotein. Psychopharmacology (Berl) 187:415–423. https://doi.org/10.1007/s00213-006-0437-9

10. Wang E, Casciano CN, Clement RP, Johnson WW (2001) EVALUATION OF THE INTERACTION OF LORATADINE AND DESLORATADINE WITH P-GLYCOPROTEIN. Drug Metab Dispos 29:1080–1083

11. Klinkhammer W, Müller H, Globisch C, Pajeva IK, Wiese M (2009) Synthesis and biological evaluation of a small molecule library of 3rd generation multidrug resistance modulators. Bioorganic Med Chem 17:2524–2535. https://doi.org/10.1016/j.bmc.2009.01.072

12. Pick A, Müller H, Wiese M (2008) Structure-activity relationships of new inhibitors of breast cancer resistance protein (ABCG2). Bioorganic Med Chem 16:8224–8236. https://doi.org/10.1016/j.bmc.2008.07.034

13. Wandel C, Kim R, Wood M, Ch MBB, Wood A (2002) Interaction of Morphine, Fentanyl, Sufentanil, Alfentanil, and Loperamide with the Efflux Drug Transporter P-glycoprotein. PubMed 96:913–920

14. Weiss J, Dormann SMG, Martin-Facklam M, Kerpen CJ, Ketabi-Kiyanvash N, Haefeli WE (2003) Inhibition of P-glycoprotein by newer antidepressants. J Pharmacol Exp Ther 305:197–204. https://doi.org/10.1124/jpet.102.046532

15. Pauli-Magnus C, Rekersbrink S, Klotz U, Fromm MF (2001) Interaction of omeprazole , lansoprazole and pantoprazole with P-glycoprotein. Naunyn-Schmiedeberg’s Arch Pharmacol Vol 364:551–557. https://doi.org/10.1007/s00210-001-0489-7

16. Zhu HJ, Wang JS, Markowitz JS, Donovan JL, Gibson BB, DeVane CL (2007) Risperidone and paliperidone inhibit P-glycoprotein activity in vitro. Neuropsychopharmacology 32:757–764. https://doi.org/10.1038/sj.npp.1301181

17. Müller H, Pajeva IK, Globisch C, Wiese M (2008) Functional assay and structure-activity relationships of new third-generation P-glycoprotein inhibitors. Bioorganic Med Chem 16:2448–2462. https://doi.org/10.1016/j.bmc.2007.11.057

18. Saleh MAA, Loo CF, Elassaiss-Schaap J, De Lange ECM (2021) Lumbar cerebrospinal fluid-to-brain extracellular fluid surrogacy is context-specific: insights from LeiCNS-PK3.0 simulations. J Pharmacokinet Pharmacodyn 48:725–741. https://doi.org/10.1007/s10928-021-09768-7
